# Patterns of Attention and Anxiety in Predicting Arithmetic Fluency among School-Aged Children

**DOI:** 10.3390/brainsci12030376

**Published:** 2022-03-11

**Authors:** Lars Orbach, Annemarie Fritz

**Affiliations:** 1 Department of Psychology, Federal University Minas Gerais, Belo Horizonte 31270-901, Brazil; 2 Academy Wort and Number, 50996 Cologne, Germany; fritz-stratmann@uni-due.de; 3 Department of Psychology, University Duisburg-Essen, 47057 Duisburg, Germany

**Keywords:** anxiety, math anxiety, sustained attention, math performance, primary school children, latent profile analysis, choking under pressure

## Abstract

Although the interaction between anxiety and attention is considered crucial for learning and performance in mathematics, few studies have examined these cognitive and affective predictors in a single framework or explored the role of sustained attention in promoting children’s arithmetic performance, using traditional linear analyses and latent profile analysis (LPA). In this paper, state anxieties (in a math test and in an attention test situation), general anxiety traits, sustained attention (performance-based test and attention deficit/hyperactivity disorder (ADHD) self-ratings) and math achievement of 403 fourth and fifth graders (55.8% girls) were assessed. A negative correlation between state anxiety prior to the math test and arithmetic achievements was identified, even when controlling for other non-math related state anxieties and general anxiety. Sustained attention was a strong predictor of arithmetic achievement and functioned as a moderator in the anxiety-performance link. LPA identified six distinct profiles that revealed a complex relationship with arithmetic fluency. The weakest achievement was found for a specific math anxiety subgroup. The findings highlight the important role of the interaction of anxiety and sustained attention in children’s ability to perform math and enable new conclusions about the specific nature of math anxiety. Implications for future research are discussed.

## 1. Introduction

For several decades, research on individual differences in mathematics has focused mainly on domain-general abilities and provided evidence that math builds on cognitive factors, such as executive functions (EF), working memory (WM) and attentional control [1,2,3,4,5]. Besides this considerable number of research findings, fewer studies have examined the role of sustained attention in supporting children’s math learning and achievement [6]. Several studies have merely applied short and diverse types of executive control assessments, or obtained information through observer ratings by teachers or parents, evaluating behavioral attention in children’s daily life activities [1,2,3,5,7].

Attentional deficits are highly associated with anxiety, and it is hypothesized that children with anxiety and attention problems have greater WM impairments [8]. Intensified research on children was able to relate anxiety directly to mathematics, which explained individual differences in math achievement (e.g., [9,10]). According to the attentional–control theory by Eysenck et al. [11], this anxiety–performance link is caused by deficits in the attentional–control system. Although the interaction between cognitive and affective factors, such as anxiety, is seen as crucial for explaining individual differences in mathematics, studies have been considering more recently cognitive and affective variables, mostly in separate research designs [12,13].

This study addresses this scarcity of research by assessing cognitive and affective variables within a single framework. By measuring sustained attention using a performance-based assessment and a behavioral self-rating questionnaire, and by evaluating general anxiety (GA) traits and state anxieties (in a math test situation and an attention test situation), the present study aimed at investigating the interplay of cognitive and affective predictors of math achievement. Besides traditional linear analyses, the study provides further insights into the attention–anxiety relationship by applying latent profile analysis (LPA). In contrast to variable-centered approaches, LPA can identify heterogeneous patterns of cognitive and affective factors in predicting arithmetic fluency in children.

### 1.1. Definition of Sustained Attention

In most theoretical models, attention is conceptualized as a multidimensional construct, including several interacting components [14]. These components allow an individual to select, integrate and retain information, or to handle and monitor competing stimuli or responses [15,16]. According to the neurocognitive model of attention by Posner and Peterson [17], the functions can be divided into three subsystems: altering, orienting, and executive control. Alerting is regarded as the ability to produce and sustain an optimal level of arousal to receive and process stimuli. A distinction is made between phasic alertness (a short-lived alertness, e.g., in the case of an external stimulus) and sustained attention (maintaining long-term alertness, e.g., maintaining vigilance on task requirements) [18,19]. Orienting refers to the network that allows the individual to select sensory input for prioritized processing. This process can be differentiated into two systems: an exogenous (bottom–up: attention shifts involuntarily to a sensory input) or endogenous (top–down: attention shifts intentionally to a specific sensory input) system [20,21]. Finally, executive control represents the network that includes the ability to monitor and regulate attentional processes (top–down regulation), allowing the individual to inhibit information/actions and to perform controlled responses [22]. The described components overlap with other theoretical models of executive functioning [23], such as the model by Miyake et al. [24]. Consequently, there is no universally accepted and distinct model of attentional control, leading to various operationalizations and difficulties in comparing research findings [25,26,27].

Besides diverse performance-based assessments of cognitive attention (e.g., flanker tasks, cued reaction time tasks, continuous performance tasks, and trail-making tests), behavioral ratings of inattention are often applied in research to assess sustained attention. These observer ratings are generally used to index the presence of attention deficit/hyperactivity disorder (ADHD) symptoms in daily life activities of children. The neurodevelopmental disorder is highly associated with impairments in attentional control functions [16,28,29,30].

### 1.2. Relation of Sustained Attention to Math Achievement in Children

To date, few studies have examined the relation between sustained attention and math achievement in children using performance-based assessments of sustained attention (Table S1). These studies are based on different sample compositions (e.g., age groups, group comparisons between children with different disabilities and controls), typically have small sample sizes, and investigate cognitive attention with different performance tests. Nonetheless, most studies were able to identify a stable association between cognitive attention and math achievement in all age groups from kindergarten children to late adolescents. There are some indications that this relation persists even after controlling for intelligence [31,32]. However, more complex and contradictory results have also been reported. For example, Szűcs et al. [33] analyzed various cognitive predictors of math achievement in nine-year-old students and did not find any predictive value of sustained attention on math performance, while it was a predictor of number sense. In this study, number sense was assessed using subitizing, non-symbolic, and symbolic magnitude comparisons tasks. Interestingly, number sense was not a predictor of math achievement, leading the authors to hypothesize that number sense may not be directly related to math achievement when other strong predictors are considered in the research design. Furthermore, in the study on sustained attention with the largest sample size ([34]: *n* = 129) children with math difficulties did not perform significantly poorer on the cognitive attention test, compared to controls.

Although pronounced discrepancies between ADHD observer ratings and performance-based assessments of cognitive attention [6,35] and between ADHD observer ratings and performance-based assessments of EF [30,36,37,38,39] were identified—indicating that the measures assessed different constructs—ADHD observer ratings also negatively correlate with math achievement [5]. Similar results exist for self-ratings of ADHD in children [7].

### 1.3. Relation of Anxiety to Math Achievement in Children

From numerous studies in the past decade, it has been sufficiently proven that anxiety is associated with lower academic performance [40,41,42,43,44,45,46] and has a negative impact on educational trajectories and career choices [47,48,49,50]. This anxiety–performance link is explained by avoidance behavior [51,52,53] and by impairments in the attention control system [11]. Thus, anxious individuals try to avoid encounters with testing or learning situations (behavioral anxiety reaction), resulting in fewer learning opportunities. In addition, EF resources are blocked during task processing (cognitive processes during a fear reaction), as the focus of attention shifts from task-orientated problem solving to threat-related stimuli [54].

New findings underline the importance of distinguishing between assessment approaches in research focusing on the anxiety–performance link [43,44,55,56,57]. A common distinction in these approaches is made between state and trait anxiety. State anxiety is defined as a temporary and situation-related anxiety reaction linked to an increased arousal of the autonomic nervous system and can be distinguished from the relatively enduring personality trait of anxiety [58]. Given subjective beliefs about emotions and their influence on individual’s responses in anxiety questionnaires [59,60], it is necessary to focus on state anxiety if researchers are interested in actual anxiety reactions and their impact on cognitive processes or academic achievement [56,57]. One situation-specific anxiety type is state anxiety in math-related situations (state math anxiety), which complies with the phenomenology of a specific phobia [61,62,63]. Math anxiety (MA) is associated with other forms of anxiety, but it has been found that different measures of MA correlate to a higher degree with each other than to general or test anxiety [64]. Furthermore, despite the etiological overlap between different anxiety types, specific genetic and environmental factors indicate that MA develops independently of GA [63,65].

As noted earlier, the interplay between anxiety and executive functioning has been recognized as being highly relevant for the affective drop in performance for decades [51,66]. However, the interrelationship seems to be complex [66] and therefore, the interplay is still the subject of ongoing research. One present-day research interest relates to the assumption of “choking under pressure” in mathematics. This assumption is based on the observation of individuals with higher EF resources that showed greater drops in math achievement than individuals with lower EF resources [7,67,68,69,70,71]. One explanation for this phenomenon could be seen in the strategy choice of individuals in stressful situations [72,73]. Due to cognitive interference, strategies that make higher EF demands cannot be readily used with anxiety present, and children with higher cognitive abilities tend to use these more demanding strategies [68,74]. Nevertheless, there are also findings available highlighting the greater risks for individuals with lower EF resources (e.g., [75,76,77,78]).

### 1.4. Research Questions in the Present Study

The interplay between attention control and affective factors is seen as crucial in explaining individual differences in mathematics [12]. However, there is a lack of research that considers sustained attention and anxiety variables in children within a single framework. Furthermore, few studies have used performance-based assessments of sustained attention rather than observer ratings. Therefore, one aim of the present study was to investigate the interplay of both factors on math achievement using correlation and regression analyses. To provide more insights into the interrelationships, the present study also applied LPA. Through this approach, the study attempted to identify different patterns of cognitive and affective factors in school-aged children. In contrast to correlation and regression analysis, LPA enables taking heterogenous groups into account, which might characterize specific subgroups in the general populations. An overview of the application and utility of LPA for research examining individual differences in learning and development was provided by Hickendorff et al. [79].

In the process of analyzing the interrelationships, the first aim was to investigate the correlations between the performance-based attention test, and the behavioral self-ratings of ADHD and anxiety questionnaires. Do self-ratings of ADHD—in contrast to the findings of observer ratings by teachers and parents—relate to sustained attention performances (research question 1, RQ1)? To what extent do different anxiety types relate to sustained attention (RQ2)? The next research question deals with the specific nature of state-MA. To what extent is state-MA related to math achievement when controlling for other state anxieties and GA (RQ3)? The fourth research question contrasts the predictive power of the different variables on math achievement. What magnitudes of influence do the different variables have on basic arithmetic skills (RQ4)? Based on the findings on the “choking under pressure” effect, the fourth aim was to examine whether sustained attention moderates the relation of anxiety to math achievement (RQ5).

A major goal of the study was to investigate whether there are different anxiety–attention profiles in children. Is it possible to identify distinct subgroups who show similar patterns on measures of state anxiety, GA traits, ADHD self-ratings, and sustained attention (RQ6)? If it is possible to classify distinct patterns, the relationship between them and math achievement will be investigated. The question then becomes, in what way are anxiety–attention profiles related to math achievement (RQ7)?

## 2. Materials and Methods

### 2.1. Participants

The sample consisted of 403 fourth (approx. 10-year-old) and fifth grade (approx. 11-year-old) students (55.8% girls; 126.48 ± 9.15 months old) from 14 regular comprehensive schools in the state of North Rhine-Westphalia, Germany. One entire class within each school was assessed. All participants attended regular schools and were not identified as students with special educational needs. The target group was chosen because anxiety and attention problems are highly relevant in these school grades and the applied questionnaires were validated for this age group [80,81]. The study was conducted in accordance with the human subject guidelines of the regional school law.

### 2.2. Procedure

The data were collected in class on two consecutive days. On the first day, state anxiety was assessed immediately prior to and after the math test. Afterward, the children rated their GA traits and ADHD symptoms. On the second day, children filled out the state-anxiety questionnaire immediately prior to and after the attention test. The research design included several state-anxiety variables to test the specificity of math-related anxiety responses and to distinguish state-MA from other influencing factors.

### 2.3. Materials

#### 2.3.1. Sustained Attention

**Figure 1:**
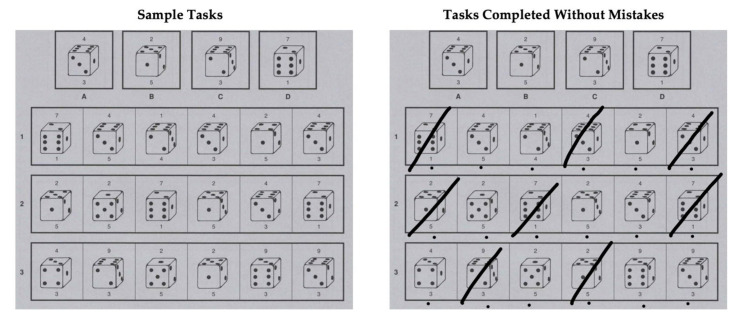
Sample tasks sustained attention test (copyright Beltz Test GmbH, Göttingen—Reprinting and any kind of reproduction prohibited).

Sustained attention was assessed with the German instrument Konzentrationstest 3-4 R (KT 3-4 R: [82]). The KT 3-4 R is a paper-and-pencil assessment that can be used in a class setting. Participants were instructed to solve as many items with low difficulty as possible within a 20 min testing period. The test includes 13 pages each with 29 items. At the top of each page, four different cubes with two one-digit numbers are presented as patterns (Figure 1). Participants were asked to complete the 29 item cubes under the patterns one by one and to compare each item cube with the patterns. Before participants responded to each item cube, they were asked to mark that cube with a dot. If the item cube and the one-digit numbers matched one of the pattern cubes, participants were instructed to cross out the item cube and to proceed to the next item. The pattern cubes changed with each page. Between the item cubes, distractors are presented, which should not be considered by the participants. The total raw score of attention was based on the subtraction of all wrong items from correctly solved items. In general, the reliability (internal consistency) of the KT 3-4 R is α = 0.87 to α = 0.92.

#### 2.3.2. State Anxiety

State anxiety was measured using the state Mathematics Anxiety Questionnaire (state-MAQ: [7]). The self-evaluation questionnaire includes seven items to assess current anxious expectation and seven items to assess state anxiety retrospectively. Participants indicate whether an emotional state applies to them currently (pre) or did so recently (post) on a 4-point-Likert scale (0 to 3). In this research project, the participants were instructed verbally and in written form to rate the items only considering the upcoming/completed math or attention test. Other circumstances were not to be considered. Immediately before the assessment, they were told that in front of them, there is a book with a variety of math/attention tasks and that they would now take a math/attention test.

#### 2.3.3. General Anxiety

GA traits were measured with the Kinder-Angst-Test-III [83]. This self-report questionnaire includes 18 items to assess a relatively enduring anxiety disposition. On a 2-point Likert-type scale (yes/no response format), children indicate whether symptoms of GA anxiety apply to them). The reliability is α = 0.88. Higher values refer to greater intensity of GA.

#### 2.3.4. ADHD Self-Rating

Children’s self-ratings of ADHD symptoms were measured with the self-rating scale for ADHD [84] from the Diagnostic System for Mental Disorders in Childhood and Adolescence. Participants indicate whether a particular symptom of ADHD applies to them on a 4-point Likert-type scale (0 to 3). The questionnaire includes nine items to assess attention deficits, seven items to assess hyperactivity and four items to assess impulsivity based on the diagnostic criteria of the 10th revision of the International Statistical Classification of Diseases and Related Health Problems (ICD). The reliability (internal consistency) is α = 0.88. Higher values refer to greater intensity of self-rated ADHD symptoms.

#### 2.3.5. Math Achievement

Six basic arithmetic operation subtests (addition, subtraction, multiplication, division, missing term, and comparison of smaller and bigger numbers) of the Heidelberger Rechentest (HRT: [85]) were used to assess math achievement. The instrument was chosen because no specific prior knowledge is required for processing the tasks, and the instrument allows arithmetic abilities to be recorded without floor or ceiling effects. Every subtest includes 40 tasks with progressively increasing difficulty. The participants were instructed to solve as many tasks as possible within two minutes (12 min in total). The total score (max. 240 points) was calculated as the sum of all correctly solved items. The reliability is α = 0.77–0.89.

### 2.4. Data Analysis

Statistical analyses were performed using R and IBM SPSS Statistics (Version 27). To examine associations between the variables, Pearson’s correlation analyses were utilized, and z-values were calculated to compare coefficients. Correlation values of *r* ≥ 0.1 were considered small, *r* ≥ 0.3 medium and *r* ≥ 0.5 large [86]. Predictors of math achievement were analyzed using a linear regression model. One-way analyses of variance (ANOVA) were performed to evaluate group differences. In line with Cohen [86], values of *d* ≥ 0.2 represent small, *d* ≥ 0.5 medium and *d* ≥ 0.8 large effect sizes, respectively, while *η*^2^ ≥ 0.01 is interpreted as a small, *η*^2^ ≥ 0.06 a medium and *η*^2^ ≥ 0.14 a large effect size. To study possible moderation effects, conditional process modelling was used by means of the PROCESS macro [87]. To identify patterns of anxiety and sustained attention, LPA was used by means of R and the tidyLPA package. Further information about the package can be found in the technical publications [88,89]. The number of profiles was determined according to the Bayesian information criterion (BIC), the sample-size adjusted Bayesian information criterion (SABIC), the bootstrap likelihood ratio test (BLRT), and the value of entropy. According to the simulation studies by Nylund et al. [90] and Tein et al. [91] BIC and BLRT are the model fit indices, which proved to be the best-performing indices for determining the number of profiles in LPA. BLRT can be used to test whether the inclusion of an additional latent profile significantly improves the model fit. Generally, an alpha level of 0.05 was applied in this study [92].

## 3. Results

Descriptive statistics (means and standard deviation) for raw values on the attention test (KT 3-4 R), the state anxiety (state-MAQ), GA traits (Kinder-Angst-Test III), self-rating scale for ADHD questionnaires, and the math achievement test (HRT) are reported in Table 1.

### 3.1. Correlation Analysis

Bivariate correlations among the variables are reported in Table 1. All anxiety variables and ADHD self-ratings were negatively associated with math achievement, while sustained attention was strongly positively correlated with math test scores. Even after controlling for ADHD self-rating, the correlation between sustained attention and math achievement remains strongly positive (*r =* 0.47). The correlation of state anxiety prior to the math test and math test scores remains significant, even after controlling for both state anxiety variables in anticipation with the attention test and GA traits (*r* = −0.0.16; *p* = 0.001), in contrast to the MA-performance link of the state MA post-test (*r* = 0.00; *p* = 0.937). Only the GA and ADHD self-rating showed small negative correlations with sustained attention. GA correlated significantly higher with ADHD self-ratings, compared to the relations of state anxieties with ADHD self-rating (*z* = 3.53 to 3.60; *p* ≤ 0.01). Bivariate correlations between anxiety and attention variables and the respective math subtests can be found in Table S2. Minor differences between the math domains were observed. Multiplication and comparison tasks had slightly lower negative correlations to the state MA pretest (*z* = −2.05 to −2.23; *p* = 0.01 to 0.02).

### 3.2. Regression Analysis

A linear regression model was calculated to examine the magnitude of influence of each variable on math achievement (Table 2). In general, the model accounted for 35% of the total variance. A strong positive predictor of math achievement was sustained attention, while state anxiety prior to the math test, GA and ADHD self-rating had negative impacts on math test scores. All other state anxiety measures were not significant predictors.

### 3.3. Moderation Analysis

**Figure 2:**
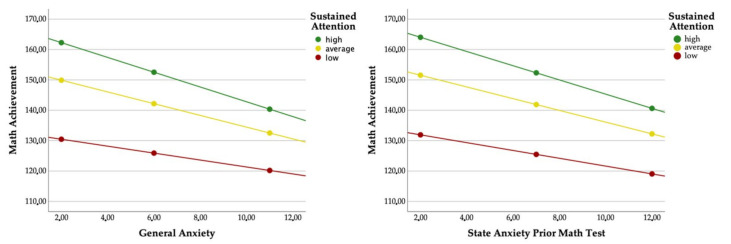
Moderation graphs (variables are plotted at 1 SD above and below M).

To analyze the effect of sustained attention on the relation between anxiety and math achievement (Table 3), moderating regressions (model 1) were applied by means of the PROCESS macro [87]. Sustained attention functioned as a moderator in the relation between state anxiety prior to the math test and math achievement as well as in the relation between GA and math achievement. Children with higher state anxiety or higher GA showed more negative relations (Figure 2). No further moderating effects could be identified.

### 3.4. Latent Profile Analysis

To identify different profiles of anxiety and attention, an LPA was applied based on scaled scores of all state anxiety measures, GA, ADHD self-rating and sustained attention. Table 4 reports the model fit information. The number of profiles was selected using BIC, SABIC, BLRT, and the value of entropy. The BIC and SABIC were minimal for the six-profile solution, while the BLRT test was statistically significant until the seven-profile solution. The entropy was 0.81 for the six-profile solution. Therefore, the six-profile model was selected as the best-fitting model.

The descriptive statistics for each profile are shown in Table 5, and all profiles are named descriptively to increase the readability:

- Profile 1: Math anxiety subgroup (high state-MA/weak sustained attention). Approximately 7% of the sample were classified to profile 1. Children in this profile reported high state-MA levels, average state anxiety in the presence of the attention test and higher GA. The subgroup showed sustained attention scores and ADHD self-ratings that differ significantly from the sample mean (*t*(29) = −3.56/6.34, *p* ≤ 0.001).
- Profile 2: GA subgroup (general high anxiety levels). About 9% of the sample belonged to profile 2, which was characterized by general higher anxiety levels and higher ADHD self-ratings. The sustained attention scores did not differ from the sample mean (*t*(36) = −1.91, *p* = 0.065).
- Profile 3: Slight tension subgroup (slightly higher state anxiety/higher sustained attention). Twenty-one percent of the sample was categorized to profile 3. Children in this subgroup reported slightly higher state anxiety levels and had high sustained attention scores. All state anxiety measures (*t*(83) = 5.04 to 16.81, *p* ≤ 0.001) and sustained attention (*t*(83) = 3.02, *p* = 0.003) differ significantly from the sample mean.
- Profile 4: Attention problems subgroup (higher GA/weak sustained attention). About 14% of the sample belonged to profile 4, which was characterized by low state anxiety measures, higher GA and weaker attention scores. Children in this subgroup exhibited lower sustained attention scores (*t*(56) = −2.01, *p* = 0.042) and reported higher ADHS self-rating (*t*(56) = 6.96, *p* ≤ 0.001).
- Profile 5: Average subgroup I (average scores). Approximately 15% of the sample was classified to profile 5, which was characterized by average scores on mostly all variables (*t*(59) = −1.11 to 1.85, *p* = 0.07 to 0.89). Only ADHD self-ratings (*t*(59) = −3.09, *p* = 0.003) differ significantly from the sample mean.
- Profile 6: Average subgroup II (lowest self-ratings). A total of 34% of the sample was categorized to the largest profile 6. Children in this subgroup reported the lowest anxiety and ADHD self-ratings, which also differ significantly from the sample mean (*t*(134) = −35.61 to −11.50, *p* ≤ 0.001). Their sustained attention scores were average to slightly higher (*t*(134) = 1.88, *p* = 0.063).

Gender ratio and age (in months) were compared across profiles and the comparisons are reported in Table 5. ANOVA revealed no differences in age between subgroups. Results of the chi-square tests of independence show a non-significant interaction between gender and profile membership (*χ*^2^(5) = 11.01, *p* = 0.51, *ϕ* = 0.17). While the gender ratio was equally distributed in profiles 1, 4, 5, 6 (*χ*^2^(1) = 0.07 to 1.20, *p* = 0.27 to 0.86), gender differences were identified in profiles 2 and 3 (*χ*^2^(1) = 3.86 to 9.76, *p* = 0.002 to 0.05).

### 3.5. Profile Comparison of Math Achievement

**Figure 3:**
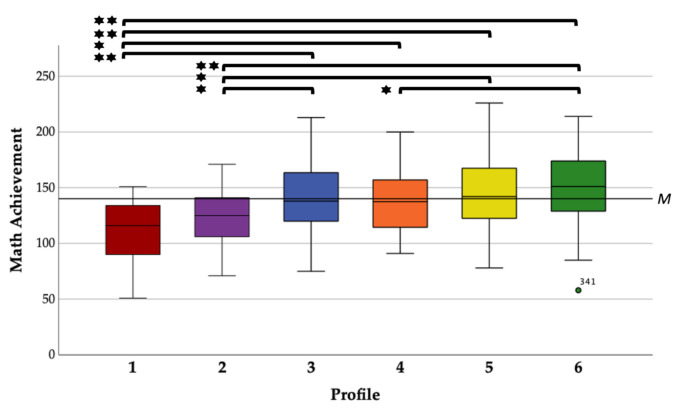
Boxplots of math achievement (total score of HRT) among LPA profiles (* *p ≤* 0.05 (2-tailed) ** *p ≤* 0.01 (2-tailed).

In Table 5 and Figure 3, math achievements are compared across LPA profiles. The one-way ANOVA reveals clear differences between the profiles. Profiles 1 and 2 showed the weakest math achievement in the sample, while profile 6 had the highest math scores. These three profiles (1, 2, and 6) differ significantly from the mean value of the whole sample (*t*(29–131) = −5.92–4.10, *p* ≤ 0.001). All other profiles showed math achievements near the sample mean (*t*(55–82) = −1.15–1.59, *p* = 0.117–0.677). In Table S3, the math subtest scores are compared across LPA profiles.

## 4. Discussion

Although the role of attention and anxiety in children’s ability to engage with mathematics is considered to be highly important, these two variables have rarely been examined in within a single framework using traditional linear analyses and LPA. Considering this absence in the current research, the aim of the present study was to examine the attention–anxiety interplay by assessing both factors with multiple assessment approaches. Attention was measured using a performance-based test and a behavioral self-rating questionnaire for ADHD, while anxiety was evaluated using a real-time paper-and-pencil assessment for state anxiety (in two test situations) and a self-report questionnaire of GA traits.

Consistent with previous data on ADHD observer ratings [6,34,35,36], pronounced discrepancies between ADHD self-ratings and performance-based assessments of attention were identified. There was only a low correlation between sustained attention and ADHD self-ratings. The attention–math performance link remains strongly positive after controlling for ADHD self-ratings. This link is also a strong predictor of arithmetic achievement. The results suggest that the two assessment approaches measure different underlying constructs. This finding underscores the importance of distinguishing between ratings of behavior and cognitive measures of attention as it is frequently assumed in the literature or in the framework of research designs [36,93].

Generally, the linear regression model was highly specific in predicting math achievement. All analyses confirmed a strong positive impact of sustained attention on math achievement. This indicates that a greater ability to maintain an optimal level of arousal to receive and process information is highly related to arithmetical achievement. In comparison to other studies on sustained attention [6,33] and executive control [1,2], these revealed correlation and regression coefficients are exceptionally high, which could be explained by the design of the attention test (e.g., tasks including digits) and the speed test design of the arithmetic test. It may be that children with higher vigilance can better manage repeated time–pressure conditions, as it was required during the six math subtests. Although all affective variables correlated negatively with arithmetic achievement, further analyses allowed a closer look at the impact of specific anxiety types. The data provided evidence that state anxiety in anticipation of the anxiety-evoking math stimulus might have the most significant and specific effect on performance. Previous research found that MA negatively relates to math achievement, even when controlling for test- and social anxiety traits [57,94]. The present study was able to compare state MA with other state anxieties and GA traits. To conclude, the data allow new assumptions about the specific nature of MA, as the state anxiety levels prior to the math test situation were the only state anxiety predictors of math achievement and were negatively related to math achievement, even after controlling for all other non-math-related affective variables. The outcomes imply that math performance-inhibiting effects might be caused by a specific effect of state MA.

In line with previous research reports [7,70,71,95], the assumption that children with higher anxiety levels should generally be impaired in their attentional control abilities is not confirmed for state MA. The anxiety–attention interrelationship seems to be more complex and should be differentiated for specific anxiety components, as GA was negatively associated with sustained attention, consistent with the results of Moran’s [66] meta-analysis. While the correlations do not clearly highlight the general influence of cognitive variables on the performance effects of anxiety, moderating regressions provide more insights into the interplay of anxiety, attention, and math achievement. Sustained attention functioned as a moderator in the relationships of state anxiety prior to the math test to math achievement as well as a moderator of GA traits to math achievement. Children with higher vigilance had more negative relations of anxiety to math achievement. Thus, the data confirm the previous findings on the state MA–math performance link [7,57,78,96]. In line with the “choking under pressure” assumption—which was previously reported in studies assessing verbal WM/central executive [68,69,71], visuospatial WM [70] and inhibition control [7] in children—children with a higher ability to maintain long-term alertness and monitor attentional processes seem to be more affected by performance-inhibiting effects caused by anxiety. Novel findings are that these moderating effects were found for general and state anxiety. More precisely, it was only state anxiety prior to the math test that functioned as a moderator. One possible explanation is that children with higher sustained attention are more vulnerable to the effects of anxiety, which can be seen in the children’s strategy selections. Children with higher cognitive abilities are more likely to use more cognitive-demanding strategies, which they cannot apply readily in pressure-evoking situations [73,97]. It may be that children with higher sustained attention rely longer on more cognitive-demanding strategies in high-pressure situations, as they are used to performing successfully in other low-pressure situations and could have less practice in adapting their approach. In contrast, children with lower cognitive abilities employ less cognitively demanding strategies [74], which they might use fruitfully in high-pressure situations [73].

To the best of our knowledge, various types of anxiety and attention have not yet been studied with an LPA. Therefore, the present study aimed to investigate, for the first time, whether there are different anxiety–attention profiles that may characterize subgroups in the general populations of school-aged children. The data revealed a complex interrelationship between the variables, underlining the necessity to take a more differentiated look at the interplay between attention and anxiety. Two profiles can be characterized as typical anxiety subgroups. Interestingly, one of them is a specific MA group with the lowest sustained attention scores that struggled the most in the arithmetic achievement test. This finding confirms the previous research, underlining the specificity of MA as a math-related phobia [61,62,63,65]. The second anxiety subgroup exhibited a generalized anxiety pattern and, intriguingly, had slightly higher state anxiety levels than the specific MA subgroup. Since their achieved math scores are by no means worse compared to the MA subgroup, this finding supports the current discussion questioning a linear MA–performance link [10,98,99,100]. It is possible that the specificity of the anxiety response is more important for the performance-inhibiting effects in mathematics than the differences between the highest levels of anxiety. Apart from that, this could also be an effect of capturing basic numerical skills. Differences between anxiety groups could occur if advanced numerical skills (e.g., problem-solving tasks) were assessed. However, for all these conclusions, further research is needed, as moderating factors, such as learning attitudes and core beliefs, may also play a role [99,101,102].

All other subgroups showed arithmetic achievement above or near the sample mean, although two other profiles showed noticeable anxiety or attention levels. One subgroup was characterized by weak attention scores and higher GA traits, while their state anxiety ratings were not notable. One could hypothesize that this is an original group of children with general attention impairments. This would be an important finding, because it highlights the key role of an interplay of state anxiety and attention in impairing arithmetic fluency. Finally, the data provided evidence for a subgroup with children who have high attention abilities and slightly higher generalized state anxieties. In line with the Yerkes–Dodson law, we can expect these children to achieve higher math scores, but the profile is a group with average performance. Perhaps they suffer an affective drop from above-average math achievement, or perhaps these children improve their attention performance by becoming more aroused through anxiety. Interestingly, a previous LPA on MA [100] identified a similar profile that had high state anxiety levels, average math core beliefs and average math achievement. Therefore, the reasons for these outcomes require further investigations.

Some implications can be derived from these findings for clinical, educational and research practice. Firstly, the reported results underscore the importance to differentiating between assessment approaches (e.g., different underlying constructs of ADHD behavior ratings and attention performance test; timing of the use of state questionnaires; and identification of specific anxiety-provoking stimuli) to obtain more valid diagnostic clarifications. Similar conclusions can be made for the development of interventions for children with anxiety and/or attentional problems. The findings underpin the need for holistic diagnostic analyses to address the complexity of the attention–anxiety relationship. Perchance, children with attentional deficits would benefit from anxiety interventions (e.g., emotion regulation strategies for test situations) to improve their academic learning and performance, whereas children with anxiety problems might benefit also from self-management [103] interventions and a more adaptive use of problem-solving strategies [104].

The applied study approach has strengths but also some limitations, which point to directions for future research. For example, the cross-sectional study was conducted on a specific age group. Consequently, the design does not allow conclusions about bidirectional connections and—in relation to developmental changes of predictive power [105,106,107,108]—other age groups. Therefore, it becomes apparent that the longitudinal data of cognitive and affective variables are necessary to gain an in-depth understanding of reciprocal effects. Secondly, the research design included several self-report questionnaires, which involves the risk of common-method variance. Future studies could try to replicate the findings by a multimethod design. Furthermore, the applied paper-and-pencil assessment is not a direct approach to measuring the math-specific arousal of the autonomic nervous system. Future research could use physiological measures or include trait-assessments of MA as a control variable. In addition, a computerized assessment of sustained attention could offer the possibility to record variance in response behavior. Finally, assessing attention with tasks involving digits could impact children who have difficulties with MA or math in general. Therefore, future studies should compare sustained attention tasks with and without digits to evaluate their effects.

## 5. Conclusions

To conclude, the present study reveals novel insights into the attention–anxiety relationship by applying linear analyses and LPA. It underlines the crucial and complex role of the interplay of sustained attention and anxiety in children’s ability to do math in high-pressure conditions. The findings support the specific nature of MA and are consistent with the “choking under pressure” assumption that children with higher sustained attention abilities may be more affected by anxiety. Directions for future research could include other math domains and test situations to gain a more holistic view on the effects of sustained attention and anxiety. Furthermore, longitudinal data are necessary to examine the development and interplay of the two math predictors.

## Figures and Tables

**Table 1:** Descriptive statistics and bivariate correlation.

Variable	M (SD)	Correlations						
		2.	3.	4.	5.	6.	7.	8.
1. State Anxiety Prior Math Test	6.86 (4.7)	0.70 **	0.67 **	0.56 **	0.40 **	0.30 **	−0.07	−0.30 **
2. State Anxiety Post Math Test	7.55 (5.3)	-	0.65 **	0.67 **	0.44 **	0.30 **	−0.02	−0.18 **
3. State Anxiety Prior Attention Test	5.19 (4.5)	-	-	0.78 **	0.43 **	0.29 **	−0.06	−0.25 **
4. State Anxiety Post Attention Test	5.17 (5.1)	-	-	-	0.43 **	0.28 **	−0.03	−0.15 **
5. General Anxiety	6.30 (4.2)	-	-	-	-	0.57 **	−0.17 **	−0.32 **
6. Self-Rating ADHD	17.42 (9.9)	-	-	-	-	-	−0.17 **	−0.31 **
7. Sustained Attention	43.06 (19.3)	-	-	-	-	-	-	0.49 **
8. Math Achievement	140.17 (31.4)	-	-	-	-	-	-	-

Note. ** *p* ≤ 0.01 (2-tailed).

**Table 2:** Linear regression model of math achievement.

	B	*SE* B	ß	*p*	*R* ^2^	*F*
					0.354	30.24
1. State Anxiety Prior Math Test	−1.49	0.42	−0.22	**0.001**		
2. State Anxiety Post Math Test	0.50	0.39	0.09	0.198		
3. State Anxiety Prior Attention Test	−0.94	0.50	−0.14	0.063		
4. State Anxiety Post Attention Test	0.77	0.43	0.12	0.076		
5. General Anxiety	−0.88	0.40	−0.12	**0.030**		
6. Self-Rating ADHD	−0.40	0.16	−0.13	**0.013**		
7. Sustained Attention	0.71	0.07	0.43	**≤0.001**		

Note. Significant values are in bold type.

**Table 3:** Moderated regression analysis of math achievement with the predictor anxiety and the moderating variable sustained attention.

Model	B	*SE* B	*t*	*p*	*R* ^2^
State Anxiety Prior Math Test × Sustained Attention	−0.03	0.02	−10.99	**0.048**	0.31
State Anxiety Post Math Test × Sustained Attention	0.01	0.01	0.55	0.581	0.26
State Anxiety Prior Attention Test × Sustained Attention	−0.01	0.02	−0.88	0.377	0.29
State Anxiety Post Attention Test × Sustained Attention	0.01	0.01	0.35	0.730	0.26
General Anxiety × Sustained Attention	−0.04	0.02	−20.14	**0.033**	0.30

Note. Significant values are in bold type.

**Table 4:** Model fit indices for latent profile solutions.

	LL	BIC	SABIC	BLRT *p*-Value	Entropy *R*^2^
1-Profile	−0.3999	0.8083	0.8038	≤0.001	1
2-Profile	−0.3617	0.7366	0.7296	≤0.001	0.86
3-Profile	−0.3443	0.7066	0.6971	≤0.001	0.89
4-Profile	−0.3386	0.7001	0.6880	≤0.001	0.85
5-Profile	−0.3360	0.6996	0.6850	≤0.001	0.83
**6-Profile**	**−0.3339**	**0.7003**	**0.6831**	**≤0.001**	**0.81**
7-Profile	−0.3330	0.7032	0.6836	0.139	0.79

Note. Bold test indicates information criteria best fit. LL, log likelihood; BIC, Bayesian information criterion; SABIC, sample-size adjusted Bayesian information criterion; BLRT, bootstrapped likelihood ratio test.

**Table 5:** Model fit indices for latent profile solutions.

Variable	M (SD)							ANOVA			Post-hoc (Scheffé)
	Profile 1 *n* = 30 (7.4%)	Profile 2 *n* = 37 (9.2%)	Profile 3 *n* = 84 (20.8%)	Profile 4 *n* = 57 (14.1%)	Profile 5 *n* = 60 (14.9%)	Profile 6 *n* = 135 (33.5%)	Overall *n* = 403	*F* (5, 397)	*p*	*η* ^2^	
1. State Anxiety Prior Math Test	12.31 (2.9)	14.46 (3.4)	8.90 (3.5)	4.89 (3.1)	7.33 (2.8)	3.24 (2.4)	6.96 (4.7)	123.43	<0.001	0.609	2 = 1 > 3 = 5 > 4 > 6
2. State Anxiety Post Math Test	11.47 (3.2)	16.24 (3.4)	10.58 (3.6)	5.28 (3.3)	8.42 (3.6)	2.98 (2.8)	7.55 (5.3)	134.72	<0.001	0.629	2 > 1 = 3 > 5> 4 > 6
3. State Anxiety Prior Attention Test	6.30 (3.1)	13.86 (4.0)	8.33 (2.7)	3.51 (2.2)	4.98 (2.3)	1.41 (1.9)	5.19 (4.5)	178.95	<0.001	0.693	2 > 3 > 1 = 5 = 4 > 6
4. State Anxiety Post Attention Test	3.80 (2.5)	15.78 (2.8)	9.55 (2.4)	2.86 (2.4)	4.85 (2.2)	0.92 (1.4)	5.17 (1.4)	377.80	<0.001	0.827	2 > 3 > 5 > 1=5 > 6
5. General Anxiety	11.07 (2.6)	10.89 (3.4)	7.38 (2.5)	9.86 (2.8)	4.02 (2.4)	2.81 (2.4)	6.30 (4.2)	114.78	<0.001	0.591	1 = 2 = 4 > 3 > 5 = 6
6. Self-Rating ADHD	27.77 (8.9)	24.03 (10.4)	19.27 (9.3)	23.84 (7.0)	14.25 (8.0)	10.86 (6.6)	17.42 (6.6)	42.75	<0.001	0.350	1 = 2 = 4 = 3 > 5 = 6; 1 > 3
7. Sustained Attention	31.43 (17.9)	37.70 (17.1)	48.55 (16.7)	37.74 (19.3)	42.68 (21.2)	46.12 (18.9)	43.06 (18.9)	6.06	<0.001	0.071	3 = 6 = 5 = 4 = 2 = 1; 6 > 1; 3 > 4; 3 > 1
Age in months	127.03 (8.8)	127.33 (9.0)	126.57 (12.3)	128.02 (7.8)	125.53 (7.2)	125.92 (8.2)	126.48 (9.1)	0.96	0.699	0.008	1 = 2 = 3 = 4 = 5 = 6
Gender ratio (percentage girls)	60.0%	75.7%	60.7%	56.1%	45.0%	51.1%	55.8%	-	-	-	
Math Achievement	112.13 (26.0)	122.05 (27.3)	141.63 (31.8)	134.79 (25.3)	144.68 (30.1)	150.98 (30.3)	140.17 (31.4)	12.56	<0.001	0.138	1 = 2 = 4 = 3 = 5 = 6; 1 < 3, 4, 5, 6 2 < 3, 5, 6 4 < 6;

Note. Only additional significances in the post-hoc test are reported that do not correspond to the general order.

## Data Availability

The data presented in this study are available on request from the corresponding author. The data are not publicly available due to privacy or ethical restrictions.

## Supplementary Materials

The following are available online at https://www.mdpi.com/article/10.3390/brainsci12030376/s1, Table S1: Previous research on sustained attention and math performance in children.; Table S2: Bivariate correlations between anxiety, attention variables and subscales of the mathematical test.; Table S3: Math subtests scores in each LPA profile.
