# Supplementary material for: Patterns of Attention and Anxiety in Predicting Arithmetic Fluency among School-Aged Children
Source: Brain Sci. 2022 Mar 11;12(3):376. doi: 10.3390/brainsci12030376 (PMC8946815; doi:10.3390/brainsci12030376)
Supplement: Supplementary file 1 [file brainsci-12-00376-s001.zip › brainsci-1594126-supplementary.pdf]

Table S1. Previous research on sustained attention and math performance in children.

	Groups	Measure Cognitive Attention	Measure Math Achievement	Relation Attention to Math Performance
Lindsay et al., 2001 (n = 83; grade 5 - 8)	Dyscalculia (n = 27) vs. Control (n = 56)	Conners' Continuous Performance Test (CBT: approx. 14 minutes):<br><br>*Scales*: omission errors; commission errors; mean response time (RT); SE of RT; mean response time<br><br>Laptop assessment	Different Assessments conducted by schools: Iowa Test of Basic Skills; Stanford Achievement Test; Missouri Mastery Test	Dyscalculia group had more omission errors and were more inconsistent on SE of RT (no gender effects)<br><br>SE-RT and percent commission errors were predictors of math performance
Huckeba et al., 2008 (n = 64; 8 - 16 years old)	Children with Tourette Syndrome (TS) (n = 47) vs. Control (n = 17)	Test of Variables of Attention (TOVA: approx. 21 minutes):<br><br>*Scales*: commission errors; omission errors; RT<br><br>Laptop assessment	Standardized arithmetic test<br><br>Two experimental calculation tasks	IQ and TOVA scores were predictors of math performance (TS group)<br><br>Group of TS with highest attentional deficits had lowest math performance on standardized and experimental tasks
Steele et al., 2012 (n = 83; 3 – 6 years old)	-	CBT (approx. 4 minutes):<br><br>Laptop assessment	"give-a-number" (Wynn, 1990)<br><br>Test of Early Mathematics Ability III (TEMA-III)	Selective-sustained attention was associated with basic numeracy and a predictor of basic numeracy (12 months after the attention assessment/longitudinal outcome)<br><br>Executive attention was associated with cardinality understanding and a predictor of cardinality, nonverbal addition/subtraction (at the time of attention assessment)
Anobile et al., 2013 (n = 68; 8 – 11 years old)	-	Visual sustained attention test (30 trials of multiple object tracking)<br><br>*Scales*: altering; Orienting; conflicting<br><br>Computer assessment	Standardized Italian Battery (Biancardi & Nicoletti, 2004)	Attentional Performance was associated with math performance (r = .40), even significant after controlling for age, intelligence, gender, reading performance

Szűcs et al., 2014 (n = 98; grade 3 -4)	-	Attend to stimuli stream of letters and to detect a target sequence (80 trials of 3 types)<br><br>*<u>Scales</u>*: number of hits; misses of targets; RT for target hits; correct rejections; false alarms for deceivers and non-target trials<br>Computer assessment	Mathematics Assessment for Learning and Teaching test<br><br>WIAT-II (numerical operations subtest)	Sustained attention had no strong relation to math performance (*r* = .21)<br><br>Sustained attention was no predictor of math achievement, but of number sense variables. Number sense was no predictor of math achievement.
Richard et al., 2018 (n = 24; 8 - 18 years old)	Survivors of lymphoblastic leukemia (n = 24)	IVA measure of sustained attention and response control<br><br>*<u>Scales</u>*: omission errors; RT of correct responses; SE of RT correct responses; commission errors; RT of all responses; stamina<br><br>Computer assessment	WJ Calculation	Visual response control and visual attention explained 26% of the variance (math performance) after controlling for IQ

Table S2. Bivariate correlations between anxiety, attention variables and subscales of the mathematical test.

Bivariate Correlations						
Variables	Addition	Subtraction	Multi-plication	Division	Missing Term	Comparison
State Anxiety Prior Math Test	-.28**	-.28**	-.19**	-.22**	-.23**	-.19**
State Anxiety Post Math Test	-.14**	-.15**	-.11**	-.16**	-.15**	-.09
State Anxiety Prior Attention Test	-.17**	-.22**	-.16**	-.22**	-.22**	-.14**
State Anxiety Post Attention Test	-.12*	-.14**	-.09	-.15**	-.11*	-.08
General Anxiety	-.28**	-.28**	-.25**	-.24**	-.28**	-.20**
Self-Rating ADHD	-.26**	-.27**	-.30**	-.32**	-.30**	-.17**
Sustained Attention	.36**	.38**	.45**	.40**	.45**	.46**

Note. * $p \leq .05$ (2-tailed) ** $p \leq .01$ (2-tailed)

Table S3. Math subtests scores in each LPA profile.

Variable	M (SD)							ANOVA		
	Profile 1<br>n = 30<br>(7.4%)	Profile 2<br>n = 37<br>(9.2%)	Profile 3<br>n = 84<br>(20.8%)	Profile 4<br>n = 57<br>(14.1%)	Profile 5<br>n = 60<br>(14.9%)	Profile 6<br>n = 135<br>(33.5%)	Overall<br>n = 403	*F*(5, 397)	*p*	η^2
Addition	23.10 (3.9)	24.57 (3.9)	26.84 (5.4)	26.09 (4.6)	27.62 (4.6)	28.20 (5.3)	26.82 (5.1)	7.64	<.001	.088
Subtraction	21.33 (4.9)	23.05 (5.3)	25.70 (6.1)	24.90 (4.5)	26.80 (5.5)	27.44 (5.9)	25.76 (5.8)	8.64	<.001	.098
Multiplication	18.20 (5.6)	21.27 (5.7)	23.76 (6.5)	22.39 (5.7)	23.50 (5.9)	25.00 (6.6)	23.30 (6.4)	7.27	<.001	.084
Division	15.70 (8.6)	16.81 (8.4)	21.79 (8.3)	20.63 (7.7)	22.30 (7.6)	23.71 (8.4)	21.43 (8.5)	7.67	<.001	.088
Missing Term	9.60 (5.5)	11.49 (4.6)	14.78 (5.7)	12.84 (5.5)	15.12 (5.8)	16.03 (5.8)	14.29 (5.9)	9.72	<.001	.109
Comparision	24.20 (5.9)	24.87 (6.2)	27.56 (7.9)	26.81 (5.6)	28.27 (5.6)	28.87 (7.5)	27.50 (7.2)	3.56	=.004	.043
